# Exhaled biomarkers in childhood asthma: old and new approaches

**DOI:** 10.1186/s40733-018-0045-6

**Published:** 2018-08-07

**Authors:** Valentina Ferraro, Silvia Carraro, Sara Bozzetto, Stefania Zanconato, Eugenio Baraldi

**Affiliations:** Women’s and Children’s Health Department, University of Padova, Padova, Italy

## Abstract

**Background:**

Asthma is a chronic condition usually characterized by underlying inflammation. The study of asthmatic inflammation is of the utmost importance for both diagnostic and monitoring purposes. The gold standard for investigating airway inflammation is bronchoscopy, with bronchoalveolar lavage and bronchial biopsy, but the invasiveness of such procedures limits their use in children. For this reason, in the last decades there has been a growing interest for the development of noninvasive methods.

**Main body:**

In the present review, we describe the most important non-invasive methods for the study of airway inflammation in children, focusing on the measure of the fractional exhaled nitric oxide (feNO), on the measure of the exhaled breath temperature (EBT) and on the analysis of both exhaled breath condensate (EBC) and exhaled air (Volatile Organic Compounds, VOCs), using targeted and untargeted approaches. We summarize what is currently known on the topic of exhaled biomarkers in childhood asthma, with a special emphasis on emerging approaches, underlining the role of exhaled biomarkers in the diagnosis, management and treatment of asthma, and their potential for the development of personalized treatments.

**Conclusion:**

Among non-invasive methods to study asthma, exhaled breath analysis remains one of the most interesting approaches, feNO and “-omic” sciences seem promising for the purpose of characterizing biomarkers of this disease.

## Background

Asthma is a common, potentially severe chronic disease that in the majority of cases can be treated effectively to control the symptoms and minimize the risk of flare-ups (exacerbations). It usually involves chronic airway inflammation [1]. Asthma affects about 300 million individuals worldwide, and 5–20% of school-age children in Europe [1]. Its prevalence has been increasing in the last two decades, and childhood asthma has become a serious public health problem because of its morbidity and related healthcare costs. The main pathophysiological features of asthma are bronchial obstruction (due to bronchial muscle constriction, mucosal edema and excessive airway secretions) and airway inflammation, but its underlying pathogenic mechanisms have yet to be fully characterized [2–5]. Indeed, it is nowadays well known that the term asthma is applied to an heterogeneous group of conditions, which are characterized by fixed or labile airflow limitation, by different patterns of inflammation, by different contribution from bacterial and viral infections, by varying degrees of cough reflex and of mucus hypersecretion [6].

A number of pathogenic factors have been identified for this complex syndrome (asthma), including genetic predisposition and several environmental factors. Also early-life events may have a close link to the development of respiratory diseases throughout the lifespan [2–5]. Viral infections, exposure to tobacco smoke, and nutritional factors are just some of the early environmental noxae that can have a role in the development of asthma and that may orient the search for new strategies for the early prevention of this condition [7–9].

The key to the disease’s pathogenesis lies in the interaction between the host and the environment, which gives rise to different clinical phenotypes with different wheezing patterns (early, transient, persistent, late-onset), different types of airway inflammation (eosinophilic, neutrophilic, paucicellular), and a different response to treatment [10]. Although these phenotypes are usually clinically relevant, they do not necessarily offer any insight about the underlying disease processes. That is why the concept of asthma endotypes has recently been introduced, paving the way to the classification of asthma in subtypes depending on the underlying functional and pathophysiological mechanisms [11]. This approach seems promising for the purpose of improving our understanding of the disease’s pathogenesis [12, 13]. In this setting, it is fundamental to search for biomarkers capable of orienting the diagnosis, management and treatment of asthma, and possibly facilitating the development of personalized treatments [14]. This could lead to a new precision medicine type approach, which firstly identifies the pathological process through non-invasive measures of airway inflammation rather than traditional symptoms and lung-function [6].

The gold standard for investigating airway inflammation is bronchoscopy, with bronchoalveolar lavage (BAL) and bronchial biopsy, but the invasiveness of such procedures limits their use in children [15]. Airway inflammation might feasibly be studied by analyzing sputum too, since its cell content correlates strongly with that of BAL fluids. Sputum induction is also less invasive than bronchoscopy, but sputum analysis is technically complicated and suffers from a marked variability in routine clinical use [15, 16].

**Fig. 1:**
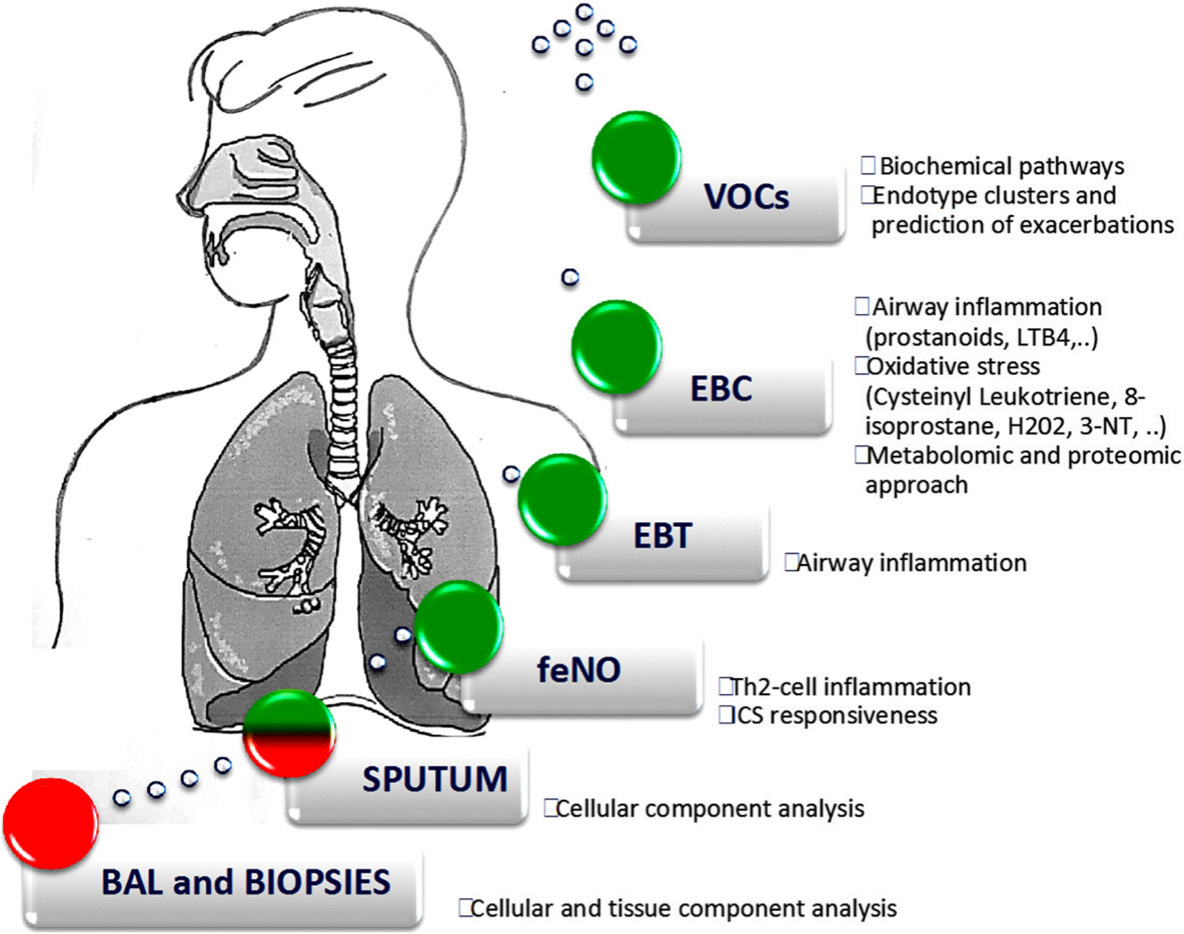
Methods for investigating airway inflammation. Red = strongly invasive, red and green = mildly invasive, and green = noninvasive

Given the drawbacks of the invasive tests available, much effort has gone into developing noninvasive methods to investigate the pathogenic mechanisms underlying asthma, based on exhaled breath analysis. Of course, the noninvasiveness of a test is particularly important when investigating childhood asthma (Fig. 1).

Here we discuss some of the methods that have been studied in recent years for their possible role in asthma characterization. The potential clinical applications and future directions for these methods are summarized in Table 1.

**Table 1:** Potential clinical application and future directions for main exhaled biomarkers

Exhaled Biomarkers	Potential Clinical Applications and Future Directions
feNO	Identification of early-onset asthma among preschool children with recurrent wheezing Stratification of asthmatic patients according to eosinophilic inflammation (treatable trait)
Analytes measured in EBC	Patient phenotyping and prediction of therapy response based on specific biomarkers profiles
VOCs in exhaled air	Early asthma diagnosis Patient stratification and phenotyping

*feNO* fractional exhaled nitric oxide, *ICS* inhaled corticosteroids, *EBC* exhaled breath condensate, *VOCs* volatile organic compounds

## Fractional concentration of exhaled nitric oxide (feNO)

The first report on the presence of gaseous nitric oxide in exhaled human breath dates from 1993 [17]. Four years later, it was found in higher than normal concentrations in children with asthma [18], and higher still during asthma exacerbations, while it dropped rapidly following oral steroid therapy [19]. As a result, the early 2000s saw a considerable number of publications exploring the relationship between fractional concentrations of exhaled nitric oxide (feNO) and asthma. Nitric oxide in the respiratory system is produced mainly by two enzymes: constitutive nitric oxide synthase (NOS), which constantly generates low concentrations of NO, and inducible NOS (iNOS), the expression of which is prompted by various inflammatory cytokines [20, 21].

FeNO can be measured using chemiluminescence and electrochemical sensors. The online single breath (SBOL) method is noninvasive, rapid, repeatable, and reproducible, and it involves the subject inhaling through the mouth for 2–3 s, to total lung capacity (TLC), then slowly exhaling immediately in conditions of velum closure (achieved by using a positive pressure of 5–20 cmH2O against the exhalation) [17, 22, 23].

The SBOL technique is well standardized and can be easily applied in children who are able to cooperate [24]. For measuring exhaled NO in young or uncooperative children, several techniques have been developed, but they have the limitation of the lack of standardization [24–26].

Several potential applications of feNO have been explored in pediatric asthma, especially as a diagnostic tool, to predict response to ICS, and to guide patient management. Many studies found a correlation between feNO and sputum eosinophilia, blood eosinophilia, serum eosinophil cationic protein, and IgE levels [17, 27]. FeNO is consequently considered a marker of a common asthma endotype characterized by Th2-mediated airway inflammation, eosinophilia, and responsiveness to inhaled steroids [17, 27]. Since increased levels of feNO have also been described in other atopic conditions, it has been suggested that low feNO levels predict a non-eosinophilic asthma phenotype better than high levels can predict an eosinophilic one [28, 29]. On the other hand, a recent systematic review supports a role of feNO in ruling in rather than in ruling out asthma [30]. In line with this assumption, it has been suggested that feNO could help us to identify early-onset asthma among preschool-age children with recurrent wheezing [31–33].

FeNO is also seen as a marker capable of predicting ICS responsiveness, since several studies found that its levels dropped significantly in response to steroid therapy [34–36]. Earlier studies on the potential role of feNO in orienting treatment decisions did not consistently find it useful to include feNO analysis in a symptom-based approach to ICS treatment, but more recent evidence suggests that it could help preventing asthma exacerbations requiring oral steroids [20]. Some authors also suggested that fluctuating feNO levels, and their cross-correlation with symptoms, can generate useful information on asthma severity and control [37]. So, even if there is no clear evidence to support the use of feNO in addition to standard symptom-based management for day-to-day asthma control [38, 39], it may be that a therapy based on feNO levels could significantly improve symptom control in some sub-phenotypes of asthma. Further studies are needed, however, based on standardized protocols and comparable study designs, before exhaled NO can be used to adjust ICS dosage and for asthma management [20].

The good standardization of the technique for FENO measurement and the studies demonstrating its potential clinical applications led to its adoption as a guide for asthma management in NICE guidelines [40]. On the other hand GINA guidelines [1] do not recommend its clinical use, yet. As recently suggested by Pavord et al. FENO could indeed have a role in the stratification of patients according to the treatable trait of eosinophilic inflammation and as a possible guide for a more personalized therapeutic approach [6].

## Exhaled breath temperature (EBT)

Exhaled breath temperature (EBT) measurement has been suggested as a noninvasive method to detect airway inflammation [41] and airway remodeling [42, 43].

Several methods have been proposed for measuring EBT, such as the rate of EBT increase, the peak of expiratory temperature (PET) and the plateau value at the end of expiration (PLET) [41, 44–47]. In pediatric population these techniques are not easy to apply, so a new and simplified device for measuring EBT during tidal breathing has been proposed [48, 49].

Previous studies demonstrated that in asthma EBT is related to the degree of airway inflammation [47], it increases in uncontrolled asthma and decreases in response to anti-inflammatory treatment [48, 50], it is significantly higher in patients with severe asthma compared to those with mild to moderate asthma [51] and it reflects changes in airway inflammation in children with virus-induced asthma exacerbations [52]. In contrast, other studies found no relationship between EBT and measures of asthma control [53].

Further studies are needed to standardize the method of EBT measurement and to better understand the usefulness of this biomarker in asthma diagnosis and monitoring in children.

## Exhaled breath condensate (EBC)

Exhaled breath condensate (EBC) can be used as a noninvasive method for studying airway inflammation. EBC is collected by cooling exhaled air by contact with a cold surface or condenser [18, 54, 55]. EBC is therefore a diluted fluid, the volume of which is almost entirely water, and consequently its analysis requires the application of highly sensitive methods for a reliable assessment of the solutes [34]. EBC collection only requires tidal breathing and it can be done safely and with no adverse effects even in preschool age. Methods for EBC collection have been developed also for very young children and infants [56].

The collected condensate contains unstable volatile (e.g. H_2_O_2_) and semi- and non-volatile molecules (proteins and cytokines) carried by respiratory droplets [54, 55]. Its composition is believed to mirror that of the airway lining fluid, thus enabling a noninvasive study of pulmonary biochemical and inflammatory processes [18, 54, 57]. Many biomolecules, markers of airway inflammation and oxidative stress, have been identified and measured in the EBC of children with asthma [57].

Although it has a great potential as a noninvasive method for measuring asthma biomarkers, the main limitation toward the clinical application of EBC remains the lack of a systematic, meticulous description of how it should be collected, preserved and analyzed. Horvath et al. [34] recently published “A European Respiratory Society technical standard”, which provides technical norms and recommendations for the collection and analysis of EBC samples. Future studies should include a systematic description of the methods used so that we can arrive at a genuine data reproducibility [34].

EBC has been studied through both a target approach (measurement of single analytes) and an untarget approach (omic techniques).

### Measurement of single analytes

Many studies investigating airway inflammation have focused on eicosanoids, a large group of heterogeneous arachidonic acid metabolites produced by free-radical or enzymatic oxygenation, including prostanoids, leukotrienes and epoxides [58]. Leukotriene (LT) B4 is a potent inflammatory mediator and a chemoattractant for neutrophils that has a role in the pathophysiology of asthma. Increased levels of LTB4 have been found in the EBC of asthmatic children. Montuschi et al. demonstrated that they were about twice as high in steroid-naïve patients with asthma as in healthy subjects [59, 60].

Cysteinyl leukotrienes (LTC4, LTD4 and LTE4) are powerful constrictors and proinflammatory mediators that have been found in higher concentrations in the EBC of patients with asthma, and particularly in cases of unstable or severe asthma [61–64]. Our research group demonstrated that Cys-LT levels dropped after a 5-day course of oral prednisone treatment for asthma exacerbations, thus showing that corticosteroids can affect the rise in LT production associated with acute asthma exacerbations [63]. Meanwhile, Bodini et al. found that exhaled Cys-LT levels and the percentage of eosinophils in induced sputum were lower after allergen avoidance [60].

As for oxidative stress, several potential biomarkers have been measured in EBC, such as 8-isoprostane, and hydrogen peroxide (H2O2). 8-isoprostane is a prostaglandin-like compound produced by arachidonic acid peroxidation. It is found in significantly higher levels in EBC from children with asthma, especially during exacerbations. A 5-day course of oral prednisone lowers the levels of 8-isoprostane, though they remain higher than in controls. This finding suggests that corticosteroids may not be fully effective in controlling oxidative stress in children with an asthma exacerbation [63]. Another marker of oxidative stress collected in EBC is hydrogen peroxide (H2O2), which derives from the inflamed airways releasing superoxide anions. A meta-analysis on asthmatic adults showed that H2O2 concentrations in EBC are higher than normal in asthmatic patients, and correlate with disease severity, disease control, and response to steroid treatment [65, 66]. They also decline in asthma patients treated with corticosteroids [66]. Similar findings in childhood asthma were reported by Jöbsis et al.: H2O2 levels in the EBC of their asthmatic patients were significantly higher than in healthy controls, and especially so in the steroid-naïve patients [67].

The products of the nitric oxide pathway (NOx), such as 3-nitrotyrosine, nitrite and nitrate, can also serve as markers of oxidative stress when measured in EBC. 3-nitrotyrosine (3-NT) derives from the nitration of the amino acid tyrosine and may serve as a biomarker of the generation of reactive nitrogen intermediates. EBC concentrations of 3-NT in asthmatic children are five times higher than in healthy controls, with no difference between steroid-naive and unstable steroid-treated asthmatic patients [68]. Two pediatric studies also found that asthmatic children had significantly higher mean nitrite/nitrate levels than their healthy counterparts, but there are conflicting results on the association between these molecules and asthma severity [69–71].

### Proteomics and metabolomics

When it comes to studying complex chronic diseases like asthma, no single biomarker can describe a full picture of the underlying pathogenic processes, but each biomarker analyzed can shed light on the mechanisms involved. On the other hand, using the “-omic” sciences enables large datasets to be obtained from single samples, potentially leading to the identification of disease biomarkers, and to the characterization of novel functional or pathological mechanisms [72]. Proteomics and metabolomics are among the “-omic” approaches applied to the study of asthma.

Proteomics involves studying the full complement of proteins in a biological sample to quantify potential biomarkers associated with a given disease [72]. Bloemen et al. used proteome analysis to study disease-specific proteolytic peptide or protein patterns in EBC samples from 30 healthy children and 40 children with asthma. The authors found a specific pattern of differentially expressed peptides in the two groups, but it is still impossible to identify individual peptides because they occur in very limited quantities and we still lack the necessary analytical sensitivity [73].

Metabolomics is an unbiased approach that, being guided by no a priori hypothesis, enables the metabolite composition of a biological sample (or metabolome) to be studied using a spectroscopic technique (usually NMR spectroscopy and mass spectrometry). The metabolome provides a picture of the state of metabolic activity that is the result of both genetic influences and environmental stimuli [74]. That is why metabolomics is considered the “-omic” science that comes closest to phenotype expression, giving us the chance to look at both genotype-phenotype and genotype-envirotype relationships [75], as well as providing a tool for studying how an organism responds to exposure to risk factors [74]. The small molecules constitute the metabolome mark fingerprints, which can be associated with phenotypes and endotypes of a given clinical condition [74]. In the study of asthma, metabolomics has been applied to several biological matrixes, including exhaled air and exhaled breath condensate.

The metabolomic analysis of EBC samples enables us to distinguish between children with and without asthma [76]. Different EBC metabolomic profiles have also been associated with different asthma phenotypes, and a particular metabolomic profile has emerged in the characterization of severe asthma [77]. Applying the metabolomic approach to blood samples also reveals a metabolic profile associated with severe asthma, including metabolites related to oxidative stress [78].

Taken together, all these studies strongly support the potential for using the -omic approaches in asthma research, though we need to consider the current limits of this approach, due largely to the lack of a standardized EBC collection method. Nonetheless, before metabolomic findings can be useful in clinical practice, they need to be replicated in multicenter studies on childhood asthma (external validation), and biomarkers identified by the untargeted metabolomic approach need to be confirmed and quantified using targeted approaches.

## Volatile organic compounds (VOCs)

VOCs originate from the lungs or upper airways and from blood circulation and they spread from the pulmonary capillary bed into the alveoli. They have been analyzed in exhaled breath using a metabolomic approach in the study of several chronic respiratory diseases, including asthma [71]. The fingerprint of VOCs in exhaled breath is called “breathome” and its study is called “breathomics” [79].

Different methods have been proposed for breath sampling. Among them the Breath Free Sampler is a highly standardized method enabling the collection of different breath volumes for a direct or offline analysis (details available at http://www.breathe-free.org). Breath Free Sampler is characterized by high repeatability and reproducibility, but it currently awaits validation in clinical trials [34].

Two different techniques have been used to study exhaled VOC profiles: (i) gas chromatography with mass spectrometry, a quantitative method that enables individual components to be identified; and (ii) the electronic Nose (e-Nose), a qualitative method that relies on a pattern-recognition technology to obtain a probabilistic discrimination between biomarker profiles [80, 81]. VOC collection is influenced by environmental, physiological and methodological factors, including conditions existing before, during and after their collection [34, 82].

Dallinga et al. found that analyzing VOCs in exhaled air using GC-MS could distinguish between children with and without asthma [83]. Other studies suggest that the analysis of exhaled VOC profiles is a promising non-invasive method for asthma diagnosis [79], monitoring [84–86], phenotyping and identification of treatable traits [87].

Pre-school children with acute respiratory wheeze have a different VOC profile compared to children with no wheezing, and such profile remains altered even after symptoms resolution in children with rhinovirus-induced wheeze [88]. Furthermore, in preschool-age children with recurrent wheezing, VOC profiles could discriminate between children with preclinical asthma and those with a transient form of wheezing, significantly improving on the prediction based on clinical data alone [89, 90].

## Conclusion

In the last 20 years, a great deal of research on the topic of asthma (particularly in children) has focused o noninvasive exhaled biomarkers. FeNO measurements cannot be routinely recommended for all children with asthma, but it could have a role in the characterization of a specific treatable trait (Th2-mediated eosinophilic inflammation). Exhaled breath analysis remains one of the most interesting approaches for studying childhood asthma, and “-omic” approaches seem promising for the purpose of characterizing biomarkers associated with specific asthma endotypes.
